# Effects of Soil Temperature and Moisture on Soil Respiration on the Tibetan Plateau

**DOI:** 10.1371/journal.pone.0165212

**Published:** 2016-10-31

**Authors:** Xiaoying Bao, Xiaoxue Zhu, Xiaofeng Chang, Shiping Wang, Burenbayin Xu, Caiyun Luo, Zhenhua Zhang, Qi Wang, Yichao Rui, Xiaoying Cui

**Affiliations:** 1 College of Life Sciences, University of the Chinese Academy of Sciences, Beijing 100049, China; 2 Key Laboratory of Adaptation and Evolution of Plateau Biota, Northwest Institute of Plateau Biology, Chinese Academy of Sciences, Xining 810008, China; 3 Key Laboratory of Alpine Ecology and Biodiversity, Institute of Tibetan Plateau Research, Chinese Academy of Sciences, Beijing 100101, China; 4 CAS Center for Excellence of Tibetan Plateau Earth Science, Chinese Academy of Sciences, Beijing 100101, China; 5 Institute of Soil and Water Conservation, Northwest A&F University, 26 Xinong Rd., 712100 Yangling, China; University of Oklahoma, UNITED STATES

## Abstract

Understanding of effects of soil temperature and soil moisture on soil respiration (Rs) under future warming is critical to reduce uncertainty in predictions of feedbacks to atmospheric CO_2_ concentrations from grassland soil carbon. Intact cores with roots taken from a full factorial, 5-year alpine meadow warming and grazing experiment in the field were incubated at three different temperatures (i.e. 5, 15 and 25°C) with two soil moistures (i.e. 30 and 60% water holding capacity (WHC)) in our study. Another experiment of glucose-induced respiration (GIR) with 4 h of incubation was conducted to determine substrate limitation. Our results showed that high temperature increased Rs and low soil moisture limited the response of Rs to temperature only at high incubation temperature (i.e. 25°C). Temperature sensitivity (Q_10_) did not significantly decrease over the incubation period, suggesting that substrate depletion did not limit Rs. Meanwhile, the carbon availability index (CAI) was higher at 5°C compared with 15 and 25°C incubation, but GIR increased with increasing temperature. Therefore, our findings suggest that warming-induced decrease in Rs in the field over time may result from a decrease in soil moisture rather than from soil substrate depletion, because warming increased root biomass in the alpine meadow.

## Introduction

Inconsistent findings about the response of soil organic matter (SOM) decomposition to warming have been reported, including positive [1, 2], neutral and insensitive [3–7] and even negative responses [8]. This discrepancy may result from complicated factors such as soil moisture, substrate availability [7, 9–15] and microbial acclimation/adaptation to warming [4, 8, 10, 16–19]. Thus, one way to improve understanding of these different effects is to conduct studies under controlled conditions [18, 20].

Previous warming studies in fields [4–8, 21] showed that initially elevated rates of soil respiration (Rs) in warmed soils returned to an even lower rate than those of the control soils. However, some results showed that warming increased substrate supply because it significantly increased dissolved soil organic carbon [22], litter decomposition rate [23], belowground biomass [24] and aboveground net primary productivity [25], suggesting that substrate depletion may not limit the response of Rs to warming. Rather, our previous results demonstrate that warming significantly reduced soil moisture in the experimental site [23, 25]. Warming experiments show that decreased soil moisture induced by warming is a likely explanation for limited positive responses of soil respiration to increased temperature (e.g. [5, 26]). Therefore, our hypothesis is that the warming-induced decrease in Rs over time may result from a decrease in soil moisture rather than from soil substrate depletion.

To test the above-mentioned hypothesis, intact soil cores with roots were taken from a warming and grazing experiment after 5-years of warming in an alpine meadow. These soils were incubated at three different temperatures with two soil moistures, and then incubated with substrate addition after 58-incubation days in our study. Our main aim was to determine the relative importance of soil moisture and substrate depletion in determining the response of soil respiration rates to warming.

## Materials and Methods

### Controlled warming-grazing experiment

Details of the experimental site and design were reported by Kimball et al. [27] and Luo et al. [23]. In brief, the experimental site is located at the Haibei Alpine Meadow Ecosystem Research Station (HBAMERS) in China, located at latitude 37° 37’N and longitude 101° 12’E. The plant community at the experimental site is dominated by *Kobresia humilis*, *Festuca ovina*, *Elymus nutans*, *Poa pratensis*, *Carex scabrirostris* and *Potentilla nivea* [25]. The soil developed is Mat-Gryic Cambisol [28], corresponding to Gelic Cambisol [29].

The warming-grazing experiment was started in May 2006. The temperature differences of the vegetation canopy between heated and corresponding reference plots were 1.2°C during daytime and 1.7°C at night in summer through infrared heaters [23, 27]. A two-way factorial design (warming and grazing) was used with four replicates of each of four treatments: no-warming with no-grazing (i.e. control-C), no-warming with grazing (G), warming with no-grazing (W), and warming with grazing (WG). In total, 16 plots of 3-m diameter were fully randomized throughout the study site. Moderate grazing was performed during the growing seasons from 2006 to 2010 [25].

### Soil sampling and analysis

On 14^th^ August 2010, ten soil cores (1.5 cm in diameter and 10 cm in depth) were randomly taken from each plot. Six of the ten cores were randomly selected to be left intact and the other four cores were first sieved through a 2 mm mesh and then through a 1 mm mesh and roots were removed at each step. All roots were collected, washed and dried at 65°C to a constant weight to measure root biomass in each plot. The six intact cores from each plot were stored in a desk refrigerator at -20°C until the incubation experiment was started to simulate the cold season in the region. Sub-samples of sieved soils were used to analyze soil moisture, total carbon and nitrogen, microbial carbon and nitrogen. Soil gravimetric moisture was obtained after oven-drying samples at 105°C for 24 hours.

Total carbon and total nitrogen were determined using an isotope ratio mass spectrometer with a Eurovector Elemental Analyzer (Isoprime-EuroEA 3000, Milan, Italy). Microbial carbon and microbial nitrogen were measured using the fumigation-extraction method with chloroform described by Vance et al. [30] and Brookes et al. [31]. In brief, fumigated and non-fumigated soils (4-g dry weight equivalent) were extracted with 20 ml of 0.5 M K_2_SO_4_ (soil/extractant ratio 1:5). The fumigation lasted for 16 h using chloroform. Samples were shaken for 1 h and filtered through a Whatman 42 filter paper. Soluble organic C and N in the fumigated and non-fumigated samples were determined using a Shimadzu TOC-VCPH/CPN Analyzer. Microbial carbon and microbial nitrogen were calculated using conversion factors of 2.64 for carbon [30] and 2.22 for nitrogen [31].

### Incubations

All frozen intact soils were thawed at 5, 15 and 25°C on 27^th^ of November 2010, and were then adjusted to 30 and 60% of water holding capacity (WHC) by adding deionized water [32]. Representative soils, treated by 1 mm sieving and drying, were used approximate soil dry weight from field-wet, bulk soils. This proportion was used to prepare one intact core from each site (approx. 10–15 g dry weight), which were incubated in 1000 ml glass bottles for 58 days in incubators set at three different temperatures (5, 15 and 25°C) (±0.4°C) and different soil moistures (30 and 60% WHC). Therefore, there were in total 96 bottles with 4 original field treatments × 3 incubated soil temperatures × 2 incubated soil moistures × 4 replicates. All bottles were opened to air without sampling.

### Glucose Addition

After 58 days of incubation, a solution containing 15 mg of glucose per gram of soil carbon was added to a 5-g (fresh wt) subsample of each soil, with the corresponding volume (1 cm^3^) of distilled water added to a further 5-g subsample [13]. All solution was directly added to the soil surface in each of the 96 bottles containing incubated cores using a 10 cc syringe with a needle tip.

During incubation, the soil cores were maintained at the designated WHC by weighing the bottles every 2 days and adding the correct amount of deionized water over the incubation experiment. Deionized water reservoirs were maintained in each incubator to allow the water additions to be made at the correct temperature. The intact cores were broken up, passed through a 1 mm sieve, and roots and stones removed at the end of the incubation. The roots and stones were oven-dried at 65°C and weighed. All sieved samples were oven-dried at 105°C to calculate the dry weight of soil in each sample.

### Respiration measurements and Q_10_ coefficients

The measurement of respiration has been described in detail by Chang et al. [32]. In brief, gas samples were taken from the headspace of the bottles using a 60 ml gas-tight syringe before being sealed with rubber stoppers and after sealed for 40 minutes by drawing and plunging the syringe three times for homogeneous gas sampling each time. Soil respiration rates were measured by the difference between accumulated CO_2_ concentrations during the 40 min incubation in the headspace of the sample glass bottles. Soils were incubated for 1 day before measurements started to allow short-term equilibration after manipulating the soil. Rs was measured during the incubation period on days 2, 9, 16, 23, 30, 37, 44, 51 and 58.

The impacts of substrate quality on short-term Rs responses to temperature were evaluated using glucose-induced respiration rates (GIR) [14]. The average respiration rate over the entire 4h incubation period serves as an index of the GIR-responsive microbial biomass pool [33] through the difference in GIR before and after glucose addition. The ratio of Rs before and after glucose addition is indicative of the carbon availability in the soil sample (e.g. Carbon Availability Index, CAI) [14]. CAI was calculated as CAI = R_Gl-_/R_Gl+_, where R_Gl-_ and R_Gl+_ are the Rs before and after glucose addition at 4h under different incubation conditions, respectively.

For all samples, CO_2_ concentration was measured by gas chromatography (HP Series 4890D, Hewlett Packard, USA) within 24 h following gas sampling. Soil respiration rates were expressed as μg CO_2_ g^-1^ soil h^-1^ [32].

Based on the Rs of different original field treatments and 3 different soil temperatures with 2 soil moistures, the Q_10_ values were calculated as Q_10_L (Low; based on the Rs measured at 5 and 15°C), Q_10_M (medium; based on the Rs measured at 5 and 25°C) and Q_10_H (high; based on the Rs measured at 15 and 25°C), with each moisture level using the average Rs at two interval temperatures (T) [34, 35]:

$$Q_{10}={\left(\frac{R_{2}}{R_{1}}\right)}^{\frac{10}{T_{2}-T_{1}}}$$

where R_1_ and R_2_ indicate the Rs on different sampling dates or the mean Rs within a certain incubation period at T_1_ and T_2_ temperature levels, respectively. The Q_10_ value was the average of Q_10_L, Q_10_M and Q_10_H for a certain treatment. We calculated the average increase in Q_10_L, Q_10_M and Q_10_H (ΔQ_10_) between before- and after- glucose addition (i.e. Gl+ and Gl- treatments) as a simple difference, i.e. Q_10_ (Gl+)-Q_10_ (Gl-) (see [14]).

### Statistical analysis

Repeated measures method of General Linear Model (SPSS 13.0, SPSS Inc. Chicago, Illinois, USA) (ANOVA) was used to assess the significance of the impacts of original treatment, incubation temperature, moisture, and incubation day, and their interactions on Rs, with original treatment, incubation temperature and moisture treated as between-subject variables and incubation day treated as a within-subject variable. A similar statistical method was used for Q_10_ during the incubation period, but omitting the temperature factor. Three-way ANOVAs were performed for the difference in Rs before- and after- glucose addition, CAI, GIR increase, glucose-inducing Q_10_ values and its change among treatments. Multi-comparisons were measured for all variables measured under different treatments when ANOVA was significant. Simple linear regression analysis was performed to test the possible dependencies of Rs on root biomass under different treatments. All significances mentioned in the text are at the 0.05 level, unless otherwise noted.

## Results

### Root biomass and soil properties

Only the original warming without grazing treatment in the field (i.e. W treatment) had significantly greater root biomass within 10 cm depth compared with C, G and WG treatments (Table 1). Generally, there were no significant differences in total carbon (8.15–8.84%), total nitrogen (0.69–0.72%), microbial carbon (1.54–1.86 g kg^-1^) or microbial nitrigen (0.49–0.51 g kg^-1^) between treatments after the 5-year warming-grazing experiment in the field (Table 1).

**Table 1:** Concentrations of total carbon (TC), total nitrogen (TN), microbial biomass carbon (MBC) and nitrogen (MBN) and root biomass in 0–10 cm soil depth under different treatments.

Treatment	TC (%)	TN (%)	MBC (g kg^-1^)	MBN (g kg^-1^)	Root biomass (g per intact core)
**C**	8.59	0.69	1.67	0.50	4.0b
**G**	8.15	0.69	1.54	0.49	4.3b
**W**	8.68	0.70	1.62	0.51	6.1a
**WG**	8.84	0.72	1.86	0.51	4.2b
**se**	0.37	0.03	0.21	0.05	0.17

Note: C: no-warming with no-grazing (i.e. control); G: no-warming with grazing; W: warming with no-grazing; and WG: warming with grazing. Different letters indicate significant differences for root biomass under different treatments. se: standard error.

### Soil respiration rate (Rs) over incubation time

**Fig 1:**
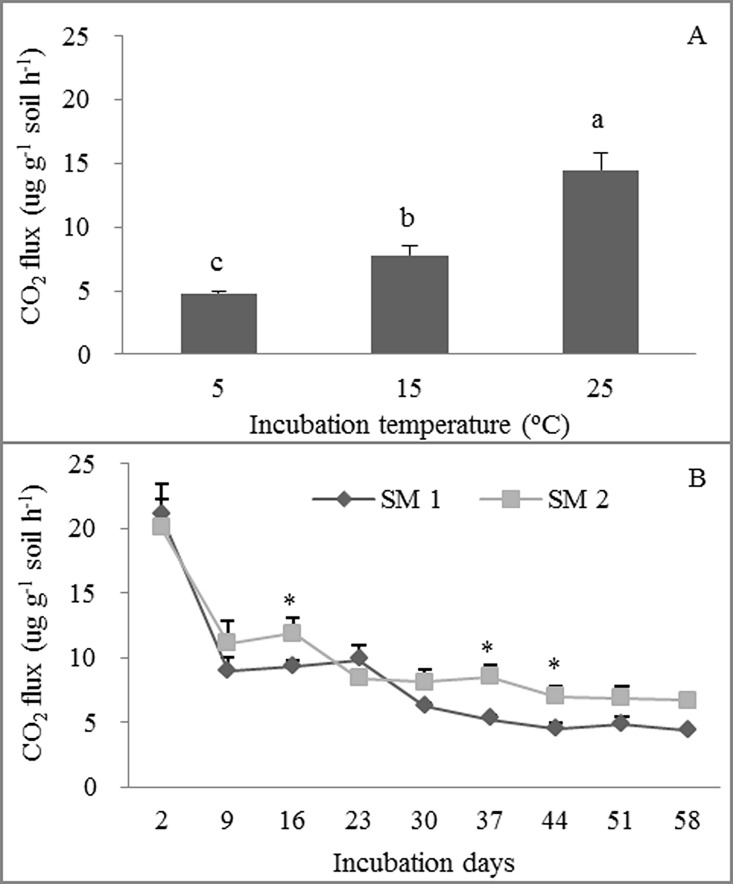
**Effects of incubation temperature (A) and incubation day (B) on soil respiration.** Mean ±se is shown in the figures. Different letters and * indicate significant difference at 0.05 level.

**Fig 2:**
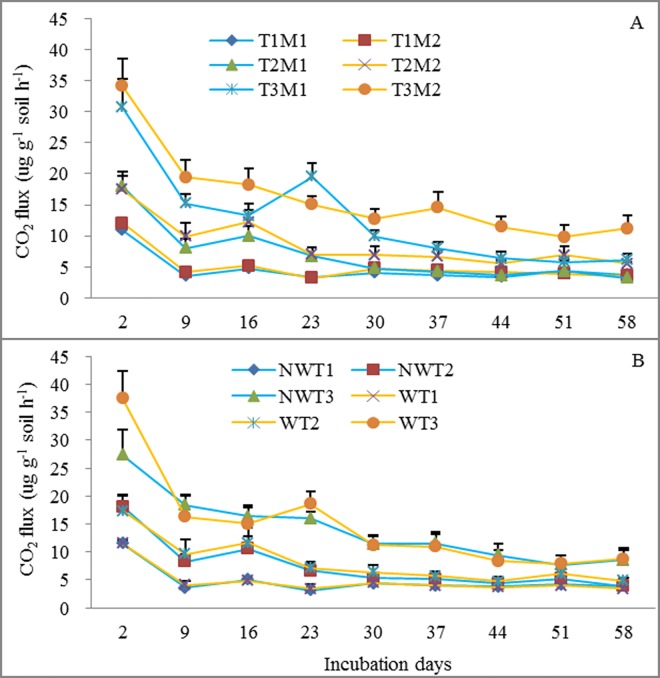
Dynamics of soil respiration over the incubation period under different treatments. (A) Combination of incubation temperature and soil moisture. (B) combination of original warming treatment and incubation temperature. T1M1: 5°C with 30% water holding capacity (WHC); T1M2: 5°C with 60% WHC; T2M1: 15°C with 30% WHC; T2M2: 15°C with 60% WHC; T3M1: 25°C with 30% WHC; and T3M2: 25°C with 60% WHC. NWT1: no-warming with 5°C incubation; NWT2: no-warming with 15°C incubation; NWT3: no-warming with 25°C incubation; WT1: warming with 5°C incubation; WT2: warming with 15°C incubation; WT3: warming with 25°C incubation; Mean±se in the figures. Different letters indicate significant difference at 0.05 level.

Soil incubation temperature and incubation day alone had significant effects on Rs, whereas the original field warming and grazing treatment alone had no significant effects on Rs (Table 2). Average Rs during the incubation period was 4.83, 7.82 and 14.52 μg CO_2_ g^-1^ soil h^-1^ at 5, 15 and 25°C, respectively (Fig 1A). An interactive effect between soil moisture and incubation day on Rs was found (Table 2 and Fig 1B). Moreover, the effects of soil moisture and the original field warming treatment on Rs varied with soil temperature and incubation day (Table 2 and Fig 2). Rs decreased with incubation days for all treatments, whereas the effect of soil moisture on Rs was significant only at 25°C (Fig 2A). Similarly, compared with the no-warming original field treatment, the original field warming treatment had significantly higher Rs only at incubation day 2 (Fig 2B).

**Table 2:** Summary of repeated-measure ANOVAs for soil respiration using original treatments, soil temperature and soil moisture incubated as main factors.

Source	df	F	Sig.
**Warming (W)**	1	0.027	0.880
**Grazing (G)**	1	1.013	0.388
**Incubation temperature (ST)**	2	41.404	**0.000**
**Incubation soil moisture (SM)**	1	5.302	0.105
**Incubation day (D)**	8	31.071	**0.000**
W * G	1	0.002	0.967
W * ST	2	0.859	0.470
G * ST	2	4.749	0.058
W * G * ST	2	2.349	0.176
W * SM	1	4.650	0.120
G * SM	1	0.991	0.393
W * G * SM	1	0.453	0.549
T * SM	2	2.964	0.127
W * ST * SM	2	2.462	0.166
G * ST * SM	2	0.043	0.958
W * G * ST * SM	2	0.317	0.740
W * D	8	2.242	0.060
G * D	8	0.706	0.684
W * G * D	8	0.181	0.991
ST * D	16	10.398	**0.000**
W * ST * D	16	2.033	**0.030**
G * ST * D	16	1.302	0.235
W * G * ST * D	16	0.725	0.755
SM * D	8	3.792	**0.005**
W * SM * D	8	2.039	0.085
G * SM * D	8	0.941	0.502
W * G * SM * D	8	0.962	0.487
ST * SM * D	16	2.747	**0.004**
W * ST * SM * D	16	0.696	0.783
G * ST * SM * D	16	1.180	0.317
W * G * ST * SM * D	16	0.595	0.872

**Fig 3:**
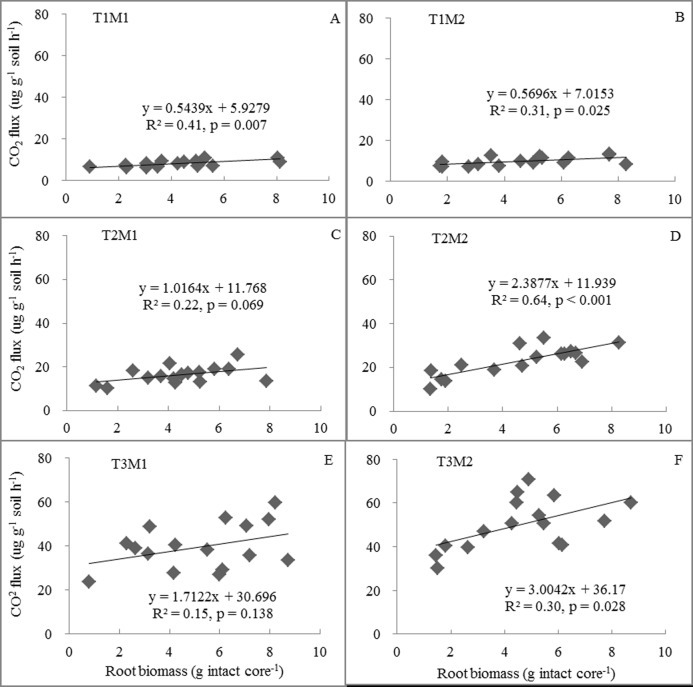
Relationships between soil respiration and root biomass under different treatments. T1M1: 5°C with 30% water holding capacity (WHC); T1M2: 5°C with 60% WHC; T2M1: 15°C with 30% WHC; T2M2: 15°C with 60% WHC; T3M1: 25°C with 30% WHC; and T3M2: 25°C with 60% WHC.

There were significant positive correlations between Rs and root biomass for all treatments except under incubation at 15 and 25°C at 30% soil moisture, and root biomass explained 30–64% of variation in Rs (Fig 3). Moreover, the dependency of Rs on root biomass increased with increase in incubation temperature at 60% soil moisture (Fig 3B–3F). Soil moisture did not affect Rs at 5°C incubation (Fig 3A and 3B), whereas it increased with soil moisture under 15°C (Fig 3C and 3D) and 25°C (Fig 3E and 3F) incubation.

### Glucose-induced Rs (GIR)

**Fig 4:**
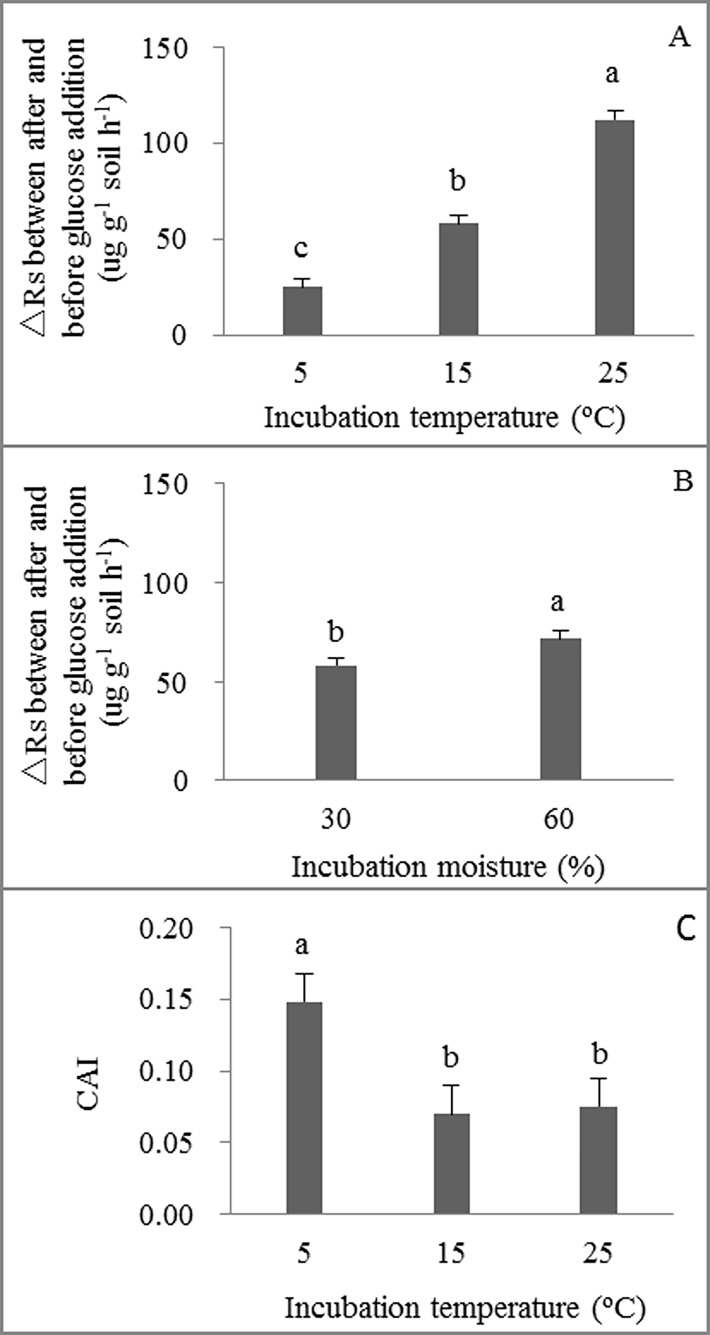
**Difference in soil respiration (μg CO**_**2**_ **g**^**-1**^ **soil h**^**-1**^**) before and after 4h of glucose addition under different incubation temperatures (A) and soil moistures (B), carbon availability index (CAI) (C) under different incubation temperatures.** Fig A and C show the mean values of 30 and 60% water holding capacity treatments under 5, 15 and 25°C incubation temperatures, and Fig B shows the mean values at 5, 15 and 25°C incubation temperature of the 30 and 60% water holding capacity treatments. Mean ±se is shown in the figures. Different letters indicate significant difference at 0.05 level.

The increased in GIR significantly increased with incubation temperature (Fig 4A) and soil moisture (Fig 4B), whereas the original field warming and grazing treatments had no significant effects on increase in GIR (*P* = 0.790). Only incubation temperature significantly affected CAI. CAI was greater under incubation at 5°C (i.e. 0.15) than at 15 and 25°C (i.e. 0.07 and 0.08, respectively) (Fig 4C).

### Rs temperature sensitivity (Q_10_)

**Fig 5:**
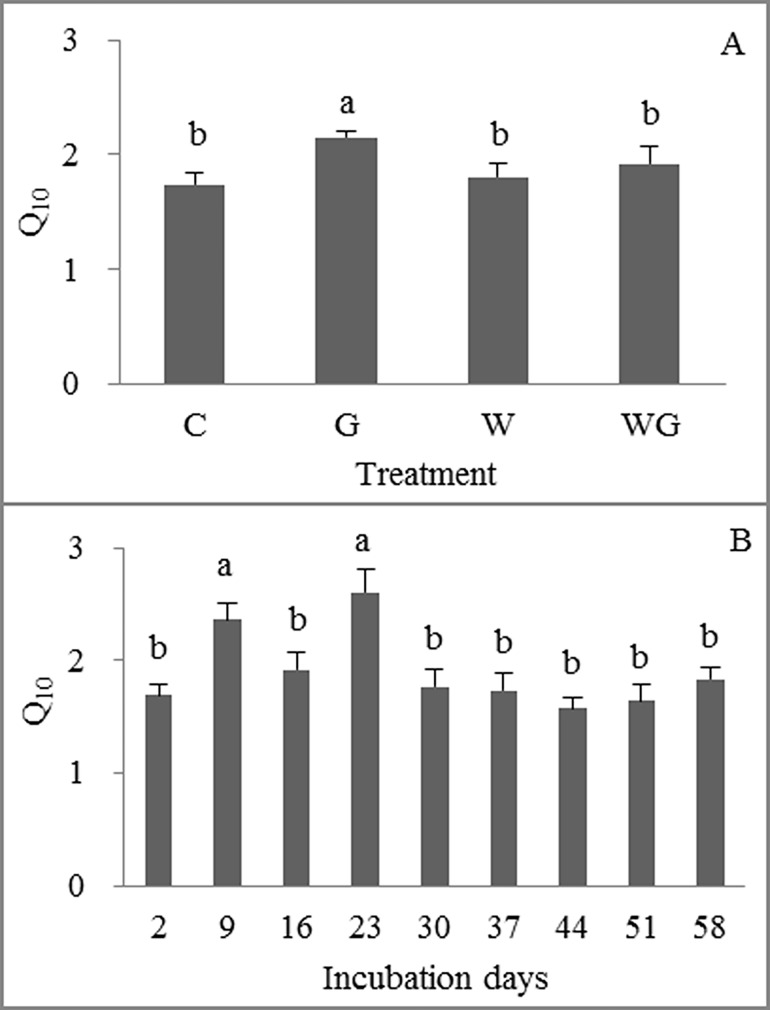
Temperature sensitivity (Q_10_) of soil respiration under different original field treatments. C: no-warming with no-grazing; G: no-warming with grazing; W: warming without grazing; WG: warming with grazing. Mean ±se is shown in the figures. Different letters indicate significant difference at 0.05 level.

The Q_10_ value was greater for the original field grazing treatment (i.e., G: no-warming with grazing) compared with the control (i.e., C: no-warming with no-grazing), warming (i.e., W: warming with no-grazing) and warming with grazing (i.e., WG: warming with grazing) treatments (Fig 5A). There were no significant differences among incubation days, except on incubation days 9 and 23, which were the highest (i.e. 2.4 and 2.6, respectively) over the incubation period (Fig 5B). Q_10_ differences before and after glucose addition were not significantly affected after 4 h of glucose addition for all treatments (data not shown).

## Discussion

Our findings indicate that the original warming and grazing treatments over 5-years in the field had little effect on Rs under all incubated soil temperature and soil moistures (Table 2), although the original field warming treatment significantly increased substrate supply to Rs through increases in plant biomass and litter decomposition rate [22–25]. CAI did not decrease with an increase in incubated temperature (Fig 4C), and lower soil moisture with greater root biomass limited the response of Rs to temperature only at higher incubated soil temperature (Fig 2). Thus, our results supported our hypothesis that warming-induced low soil moisture rather than substrate depletion may weaken the positive effect of warming on soil respiration rates in the field.

### Response of soil respiration rate (Rs) to soil temperature and moisture

Generally, we found that Rs increased with temperature, and soil moisture alone had no effect on Rs (Table 2), implying that substrate availability and soil moisture were not limitations on Rs at lower incubation temperatures (i.e., 5 and 15°C) in our study. However, previous studies in arid and semiarid ecosystems [36, 37] and in mesic systems [38] have shown that the indirect effects of warming on soil moisture can outweigh the effects of thermal stimulation on microbial activity [39]. In our study, however, at 25°C temperature 60% WHC significantly enhanced average Rs compared with 30% WHC during the incubation period, suggesting that limitation of soil moisture on Rs may occur at the higher temperature relative to 5 and 15°C (Fig 2A). Soil moisture could be a limiting factor when substrate availability (i.e. root biomass) increased at the higher temperature in our study (Fig 3). Moreover, when substrate is sufficient (i.e. with glucose addition), soil moisture had a significant effect on Rs (Fig 4B). Previous laboratory studies found that soil moisture and substrate availability affected the temperature sensitivity of Rs [40, 41]. In our study, we found that soil moisture did not significantly affect the temperature sensitivity of Rs (i.e. Q_10_), but the original field grazing treatment significantly increased Q_10_ (Fig 5A). This is probably because grazing reduced aboveground and litter biomass [23], which could decrease labile carbon in soils [10, 38]. Therefore, our results suggest that there may be a soil moisture-temperature threshold on Rs. Limitation of soil moisture on Rs may be small when environmental temperature is lower during early and late growing seasons, whereas warming-induced decrease in soil moisture may limit the response of Rs to temperature during mid-summer when environmental temperature is higher in the field.

### Response of soil respiration rate (Rs) to substrate quality

There were no significant differences in Rs between the original field warmed plots and control plots when measurements were made at a common temperature (Fig 2A), which is inconsistent with previous reports [12], although warming increased root biomass in our study, which is a contrary finding to those reported by Hartley et al. [12]. However, the dependency of Rs on root biomass increased with soil temperature and soil moisture under incubation (Fig 3), suggesting that higher Rs may stem from root decomposition and its potential priming effect on microbial respiration at higher soil temperature and higher soil moisture [12, 15, 42–44]. Glucose-induced respiration rates are usually performed to evaluate the impacts of substrate availability on short-term Rs responses to temperature [14]. If the response of Rs to temperature is suppressed due to substrate depletion [12, 45], glucose addition should increase the response of Rs to temperature. However, we found that there were no significant differences in CAIs after 58 days of incubation with glucose addition among the original field treatments when measurements were made at a common soil moisture rate (data not shown). Some empirical studies suggest that the decomposition of recalcitrant carbon is more sensitive to temperature changes than labile carbon [46–48]. However, we found that Q_10_ did not significantly decline over the incubation period (Fig 5B), suggesting further that soil substrate depletion may not limit the response of Rs to temperature in our study.

## Supporting Information

S1 Data Incubation data. (XLSX)
